# Isolation of the Flavonoid from Bamboo Residues and Its Application as Metal Ion Sensor in Vitro

**DOI:** 10.3390/polym11091377

**Published:** 2019-08-22

**Authors:** Yan Su, Huiling Dong, Min Li, Chenhuan Lai, Caoxing Huang, Qiang Yong

**Affiliations:** 1 Key Laboratory of Forestry Genetics & Biotechnology (Nanjing Forestry University), Ministry of Education, Nanjing 210037, China; 2 Jiangsu Co-Innovation Center of Efficient Processing and Utilization of Forest Resources, College of Chemical Engineering, Nanjing Forestry University, Nanjing 210037, China; 3 College of Furnishings and Industrial Design, Nanjing Forestry University, Longpan Road 159, Nanjing 210037, China

**Keywords:** flavonoids, metal ions, fluorescence sensor, antioxidant, bioimaging

## Abstract

Fluorescence sensors prepared from natural polymers have received increasing attention based on their luminescence characteristics for bioimaging, cell imaging, and intracellular detection of inorganic metabolites. In this work, flavonoids isolated from bamboo residues (BRF) were applied as fluorescence sensors for different metal cations’ detection in vitro. Results showed the optimal flavonoids extraction condition of solid to liquid ratio, ethanol concentration, extraction time and temperature were determined at 1:25, 50%, 240 min and 90 °C, respectively, resulting in an extraction yield with 104.7 mg/100 g bamboo residues. The BRF is mainly composed of isoorientin, isovitexin, pinosylvin, tricin and isorhamnetin by liquid chromatography–mass spectrometry (LC-MS) analysis. It is found that the BRF displayed strong blue-green emission as well as notable excitation, which can selectively and sensitively detect Fe^3+^ with the limit of detection (LOD) as low as 38.0 nM. In the Fe^3+^ detection was no obvious interference by other cations except for Al^3+^. In addition, the BRF displayed excellent biocompatibility that can be applied to bioimages of the intracellular detection of Fe^3+^ in L02 cells. Finally, it is found that the BRF possessed significant antioxidant properties in scavenging H_2_O_2_-induced endogenous reactive oxygen species (ROS) in a zebrafish module (in vivo) and L02 cells (in vitro). These results showed that the flavonoid products sustainably isolated from an abundant lignocellulosic waste appear to be effective fluorescent sensors for Fe^3+^ detection in biological systems with excellent biocompatibility and antioxidant activity.

## 1. Introduction

Growing societal concerns about environmental pollution have prompted demands for renewable and sustainable bio-based materials. Lignocellulosic biomasses such as forest and agriculture residues are a candidate for producing biofuels, bio-based chemicals and bio-materials because they are abundant and presently of low cost [1,2]. As one of the most important forest resources, bamboo is a particularly attractive lignocellulosic resource due to its rapid growth rate and wide range of applications [3]. Examples of applications that utilize bamboo include its use as a substitute for wood in flooring and furniture manufacturing. In the bamboo utilization industry, present technical challenges result in about 46 million tons of bamboo processing residues going unused on an annual basis in China [4]. These residues, which comprise leaves, green bamboo, and yellow bamboo, are currently (and inefficiently) burnt for energy or simply landfilled. While, these bamboo residues have been of great medical use in eastern culture due to the documented pharmacological and biological functions of their extractives [5]. Hence, efficient utilization of bamboo residues for producing biological products is the ideal choice for the valorization of bamboo residues.

Anatomically, bamboo residues comprise a complex lignocellulosic matrix containing three main polymeric constituents: cellulose, hemicellulose and lignin. The general chemical composition of bamboo residues is 40%–45% cellulose, 28%–34% hemicelluloses, and 13%–24% of lignin, as well as some flavonoids [6]. In the cell walls, cellulose is tightly associated with hemicellulose and lignin, resulting in a strong recalcitrance towards conversion of the polysaccharide polymers into fermentable sugars. Apart from these polymers, there exists a variety of flavonoids, which are fixated to the hard tissues of bamboo with partial responsibility for bamboo’s recalcitrant properties. Flavonoids are mainly in the insoluble form of free aglycone or flavonoid ligand in the hard tissues of bamboo, which make the extraction process difficult. Some general methods for flavonoid extraction include application of a heat reflux, alkaline treatment, or enzyme-assisted liberation of these compounds [7,8,9]. Recently, a variety of emerging extraction technologies involving the assistance of ultrasonic treatment have been developed for more efficient flavonoid extraction from bamboo. Compared with conventional methods, ultrasound-assisted ethanol extraction has a lowered extraction time, which both preservers flavonoid bioactivity and results in processes with greater production rates [10]. This greater efficiency is attributable to the effect of ultrasonic vibration on bamboo cells, which promotes deconstruction of cell walls and solvent penetration [11]. Based on these benefits, such methods have attracted signification attention as a viable process for extracting flavonoids from plants.

It has been reported that more than 9000 varieties of flavonoids have been identified, they exist in the form of either glycosylated or esterified derivatives with a basic 2-phenylchromanone structure consisting of C6–C3–C6 rings [12]. Classes of flavonoids include flavones, flavonols, flavanones and many more. Various classes of flavonoids different along the lines of their oxidation level and substitution pattern on their respective structure rings. Importantly, the two benzene rings in the flavonoid structure are different among the same subclass. Many studies have reported that flavonoids exhibit a variety of bioactive benefits, such as anti-viral/bacterial, anti-inflammatory, anti-cancer, and hepatoprotection [13,14,15]. An example of value-adding flavonoids is tricin, one of the dominant flavonoids in bamboo residues. It has been reported that tricin extract has cancer-treating properties, as well as the potential for aiding in the treatment of cytomegalovirus [16]. More importantly, it has been reported that flavonoids can be used as antioxidants with the capability of scavenging free radicals or chelating of metal ions in vivo [17]. Based on all of these benefits, it is certain that flavonoids are viable for application in medicines, functional foods and cosmetics.

In recent development, research has been conducted intended to investigate the feasibility of flavonoids-based natural materials as fluorescent sensors used in detection of metal ions, such as Al^3+^, Fe^3+^, Zn^2+^, Cu^2+^ [18,19]. Among these metal ions, Fe^3+^ plays an important role in many biological processes and affects human health. Excess or deficiency of Fe^3+^ can result in serious disorders involving protein damage, anemia, low blood pressure and decreased immunity [20]. Thus, effective method for determination of metal ions is of great significance to biochemical, environmental and industrial purposes. Conventional spectrophotometric, electroanalytical techniques available for metal ions detection exists the problems of time-consuming, expensive requirements and complex. So, fluorescent sensors gained considerable attention due to its high sensitivity, simple, rapid detection and low cost. Many types of fluorescence sensors currently exist, including those that are polymer-based, quantum dots, carbon nanotubes or nanoparticles [21]. Many of these sensors have demonstrated the capability to detect aqueous metal ions like Cu^2+^, Al^3+^, Mg^2+^, Zn^2+^ and Fe^3+^. However, most of them were synthesized by organic reaction with toxic reagents. A fluorescence sensor based on a Schiff compound was designed and synthesized to recognize Al^3+^ with the detection limit of 10^-7^ M [22]. Compared to these compounds, flavonoids, as natural products, is highly promising to be natural fluorescent sensors for metal ions with high sensitivity and selectivity and good biocompatibility. It had been reported that fluorescence sensors composed of flavonoids could be used to detect and bio-image metal ions within cellular structures. For example, a combination fluorescence sensor of both carbon dots and quercetin provided bio-imaging of the distribution of Zn^2+^ in HeLa cells [23]. This is a lane for the abundant flavonoids from bamboo to be used in this application as a sustainable alternative that also promotes use of a waste biomass stream. However, few works have been carried out which study development of fluorescent sensors based on flavonoids extracted from bamboo residues to detect metal ion in the biological system.

Reactive oxygen species (ROS) are chemically defined as reactive molecules including hydrogen peroxide, superoxide radical, hydroxyl radical and nitrogen-containing peroxynitrite [24]. The balance between ROS production and elimination is essential for biological process involving cell signal transduction and homeostasis. The overproduction of ROS will cause cellular damage and various diseases such as oxidative stress, cancer, inflammation and others. Hence, research has paid attention to seek the antioxidants with low-toxic and strong activity. Natural products, such as polysaccharides from different biomass, are known to inhibit H_2_O_2_-stimulated ROS generation in vivo zebrafish and in vitro vero cells [25]. However, little work has been done to investigate if the bamboo residues flavonoids can be the antioxidant in scavenging ROS in vivo and in vitro cells.

In this work, ultrasound-assisted ethanol extraction followed by purification were carried out to obtain the flavonoids from bamboo residues (BRF). Flavonoid extraction was optimized with regard to several variables involved in the method. The main component in BRF was revealed by liquid chromatography-mass spectrometry (LC-MS) technology. The fluorescence properties of the BRF were investigated and used as the probe to detected the Fe^3+^ concentration. In addition, the cell compatibility of BRF was evaluated and its intracellular bioimaging of Fe^3+^ was conducted using L02 cells. The antioxidant activity of BRF evaluated by scavenging endogenous ROS in L02 in vitro and endogenous ROS in zebrafish model in vivo. This work will serve as a demonstration of how valuable chemicals from bamboo residues can be elegantly applied to generate value for the emerging bio-economy.

## 2. Materials and Methods

### 2.1. Materials 

Moso bamboo (*Phyllostachys pubescens*) residues were provided by a bamboo processing factory in Fujian, China. Bamboo residues was pulverized using a laboratory grinder and milled into 20–80 mesh (average sizes of 0.18–0.85 mm). Milled materials with 10% moisture content was stored at room temperature until use. The samples of bamboo residues were extracted with petroleum ether for 6 h to remove lipid components and air dried for 24 h prior to the experiment.

### 2.2. Ultrasonic-Assisted Ethanol Extraction (UAEE)

Extraction conditions were investigated by employing single-factor experiments. Factors corresponding to solid-liquid ratio, ethanol-water concentration, temperature and time were investigated to better understand their effects on the yields of bamboo residues flavonoids. Six levels related to each variable were tested, with a range of 1:15–1:40 g/mL, 40%–90%, 50–100 °C, and 30–300 min, respectively. The following standard procedure was applied while changing the value of one factor at a time. Extraction was performed in a 250 mL round-bottomed flask. Specifically, samples of 3 g (on a dry weight basis) were weighed and added into the flask. 70% (*v*/*v*) ethanol solutions were poured into the flask and mixed with samples, achieving a final solid-to-liquid ratio of 1:30. Then, the flask was put under an ultrasonic cell disruptor (30 min and 500 w). After that, the flask was placed in a laboratory oil bath with magnetic stirring for 240 rpm/min. The apparatus was refluxed at 80 °C for 60 min. After extraction, the resultant liquor and solid residues were separated by vacuum filtration. Residues were washed again using fresh 70% ethanol. Extraction liquor combined with wash liquor was then kept under refrigeration (4 °C) for further use.

According to the results given by a single-factor experiment, an orthogonal experiment for four factors and three levels was designed to further optimize extraction conditions. This experiment was conducted congruent with the previously described procedures.

### 2.3. Separation and Purification of Flavonoids from Extraction Solution 

Flavonoid separation was performed using a glass column filled with wet HPD 600 macroporous resin (polystyrene, 0.3–1.2 mm particle size, Cangzhou Bon Adsorber Technology Co., Ltd., Cangzhou, China). Before separation, the resin required pretreatment through immersion into 150 mL of absolute ethanol for 4 h. After soaking, the resin was eluted with 200 mL of deionized water to remove the muddy solution generated. Water elution was continued until the aqueous eluent registered no absorption under an ultraviolet (UV) spectrophotometer. The flavonoid solution collected from best extraction conditions (150 mL) was first diluted with large amount of deionized water and then loaded through the column. After absorption, the column was eluted with deionized water (100 mL) to remove the absorbed impurities. Subsequently, the column was eluted with 200 mL 70% ethanol solution to remove the absorbed flavonoids. The eluent was collected and evaporated to solid by rotary evaporation. The solids obtained were termed bamboo residue flavonoids (BRF).

### 2.4. Determination of Total Flavonoids Content in the Extraction Solution 

Total flavonoids content was performed with a photocolorimetric method, using a rutin standard as a control. 10% Al(NO_3_)_3_ (150 mL), 5% NaNO_2_ and 4% NaOH solutions were prepared in advance. Next, a 0.15 mg/mL solution of rutin standard was prepared using the rutin standard and pure ethanol. Rutin standard solutions of different volumes in the range of 0 to 3.5 mL were separately reacted with 0.3 mL 5% NaNO_2_ solution for 6 min followed by a second reaction with 0.3 mL of 10% Al(NO_3_)_3_ solution for 6 min. The twice-reacted solution was then mixed with 2 mL 4% NaOH solution, followed by the addition of ethanol solution to reach 10 mL in total volume. After 10 min, the absorbance of these solutions were read at 510 nm using a UV spectrophotometer. Based on absorbance recorded, a linear regression between rutin concentration (C, mg/mL) and absorbance (A) was established. The amount of total flavonoids was calculated according to the following equations:

C = 0.1012A − 0.0062, *R*^2^ = 0.9954 (1)

### 2.5. The Component Analysis of Bamboo Residue Flavonoids (BRF) by Liquid Chromatography-Mass Spectrometry (LC-MS)

The BRF was analyzed by the LC-MS technology. The LC system operation conditions are: UPLC (Thermo Scientific Q Exactive Ultimate 3000 Ultra Performance Liquid Chromatography); C-18 (Accucore aQ, 2.1 × 150 mm, i.d., 2.6 μm) column; temperature: 25 °C (column); wavelength: 280 nm; mobile phase: acetonitrile+0.1% formic acid(A), water+0.1% formic acid (B); flow rate: 0.2 mL/min (gradient elution) running 50 min. Mass spectrometry system: ES (Electrospray ionization); Mass spectra mode: positive and negative; mass analysis range: 50–1500 m/z; spray voltage: 3200 V; source temperature: 350 °C; capillary temperature: 300 °C; auxiliary gas flow: 15 L/min; sheath gas flow: 40 L/min. The comparative analysis of the obtained peaks with respect to mass spectral data of standard compound(s) was used to identify known compounds in the BRF.

### 2.6. Fluorescence of BRF Solution

The bamboo residues flavonoid was dissolved into CH_3_OH/Tris-HCl buffer solution (1:99, *v*/*v*) as BRF solution for the following characterization and use, which concentration is 0.08 g/L.

#### 2.6.1. Fluorescence Emission Spectra under Various Excitation Wavelengths and Temperatures

The BRF solution used here was following another 25x dilution, which concentration is 3.2 mg/L. Fluorescence emission spectra of BRF solution was recorded at different excitation wavelengths (370–440 nm). To investigate the effect of temperature on fluorescence, BRF solution was incubated at 4, 20, 30, 37, 50 and 60 °C for 12 h. Then, fluorescence emission spectras of different temperatures were detected at an excitation wavelength of 420 nm by PerkinElmer LS55 spectrophotometer.

#### 2.6.2. Selective Detection of Different Metal Ions

Aqueous solutions containing different chloride salts with various cations (Na^+^, Mg^2+^, Al^3+^, K^+^, Ca^2+^, Mn^2+^, Fe^3+^, Zn^2+^, Co^2+^, Cu^2+^) were prepared with concentration of 4 mM. Selective detection of *Fe^3+^* was conducted in working volume of 5 mL. The BRF solution (0.2 mL, 0.08 g/L) was dissolved in 0.2 mL CH_3_OH/Tris-HCl buffer solution, subsequently mixed with different metal cation solutions (0.1 mL), respectively. After equilibrated for 2 min, Fluorescence emission spectra was recorded at an excitation wavelength of 420 nm.

#### 2.6.3. Detection of Fe^3+^

Detection of Fe^3+^ was carried out by adding different volumes of Fe^3+^ solution (4 mM) into BRF solution (0.2 mL) in total working volume of 5 mL. Addition volumes of Fe^3+^ solution varied from 20, 50, 65, 80, 100, 115, 130, 165, 180 to 200 μL. After the equilibration, fluorescence emission spectra from the test solutions were individually detected at an excitation wavelength of 420 nm. 

#### 2.6.4. Interference of Other Metal Cations on Fe^3+^ Detection

The BRF solution (0.2 mL, 0.08 g/L) was mixed with Fe^3+^ solution (0.1 mL, 4 mM) before the addition of other metal cations solution. Subsequently, 0.1 mL other metal cations solutions (Na^+^, Mg^2+^, Al^3+^, K^+^, Ca^2+^, Mn^2+^, Fe^3+^, Zn^2+^, Co^2+^) with concentration of 4 mM was separately added. Similarly, fluorescence emission spectra was recorded at an excitation wavelength of 420 nm. 

### 2.7. Cell Viability Evaluation of BRF

The 3-(4,5-dimethylthiazol-2-yl)-2,5-diphenyltetrazolium bromide (MTT) assay of L929 fibroblasts was carried out to estimate cell viability of BRF in vitro. First, fibroblasts were seeded into 96-well plates and incubated at 37 °C in an atmosphere with 5% CO_2_ for 24 h to allow the cells to attach to the wells. After the attachment period, 20 µL of MTT solution was mixed with 100 µL of BRF solution at concentrations of 25, 50, 100, 200 and 400 µg/mL. These new mixtures were further incubated at 37 °C for 4 h. After incubation, the absorbance of each well was measured at 490 nm using a microplate reader to calculate the growth of fibroblasts. All assays were performed at least in triplicate.

### 2.8. Intracellular Determination of Fe^3+^ by BRF

L02 cells (human normal hepatocyte cells) were grown in Dulbecco’s modified Eagle medium (DMEM) supplemented with 10% fetal bovine serum (FBS) for 24 h before monolayer culture. Then, medium was changed and the L02 cells were washed with phosphate buffer (PBS, pH = 7.4) for three times. The cells with different concentrations of Fe^3+^ were cultured in an incubator for 1 h at 37 °C. After incubation, the cells were co-cultured with 0.2 g/L BRF for 3 h at 37 °C. At last, the cells were washed three times with PBS for fluorescent recogniting of Fe^3+^, which were recorded by confocal laser scanning microscope.

### 2.9. Free-Radical Scavenging Activity Assay

The free radical scavenging activity of bamboo residues ethanol extracts solution was measured using its response to DPPH (1,1-diphenyl-2-picrylhydrazyl), performed in an assay based on the method in the work of Brand-Williams et al. [26]. First, 2 mL ethanol extract solution was added to 2 mL DPPH ethanol solution (0.2 M). Next, the mixture was shaken and kept in the dark for 30 min. At the conclusion of the incubation period, absorbance was read at λ = 517 nm. A mixture of 2 mL DPPH and 2 mL ethanol solution served as the blank. In addition, 2 mL ethanol extracts solution and 2 mL ethanol solution were mixed as an unreacted control. The percentage of scavenging activity was calculated as follows:

(2) $$\mathrm{Scavenging}\;\mathrm{activity}=1-\frac{A_{s}-A_{c}}{A_{b}}\times 100\%$$

where A_s_ is absorbance value of the sample, A_c_ is absorbance value of the control and A_b_ is absorbance value of the blank.

### 2.10. Antioxidant Activity of BRF on Hydrogen Peroxide-Induced Reactive Oxygen Species (ROS) Generation in In Vitro L02 Cell and in In Vivo Zebrafish 

#### 2.10.1. In Vitro L02 Cell

L02 cells were cultured in DMEM containing 10% fetal bovine serum (FBS) for 24 h following the monolayer culture. When cells grew well, they were sequently treated by fresh medium (control), H_2_O_2_ (0.25 mM) as well as H_2_O_2_ (0.25 mM) coupling BRF (0.2 g/L). After treating for 24 h, the medium was changed and the cells were washed with PBS for three times. Then, cells of three groups were incubated with oxidation-sensitive fluorescent probe dye of 2,7-dichlorodihydrofluorescein diacetate (DCFH-DA). The cell images of the all samples were recorded by using a confocal laser scanning microscopy.

#### 2.10.2. In Vivo Zebrafish 

The gamic zebrafish embryo (4 h), obtained from the Institute of Hydrobiology of the Chinese Academy of Science (Wuhan, China), were transferred to the individual wells of a 6-well plate. The zebrafish embryos were cultured by the exposed water (control group), 5 mM H_2_O_2_ (oxidative stress group) as well as mixture of 5 mM H_2_O_2_ coupling 0.2 g/L BRF (recovery group) for up to three days post fertilization (dpf). After that, cells of three groups were incubated with DCFH-DA fluorescent probe and wrapped with tinfoil to prevent the fluorescence quenching. After incubation, the embryos were rinsed in fresh embryo media and anesthetized before visualization. Fluorescence Images of embryos were photographed by a confocal laser scanning microscopy.

## 3. Results and Discussion

### 3.1. Optimization of UAEE Process for Total Flavonoids Extraction from Bamboo Residues 

**Figure 1:**
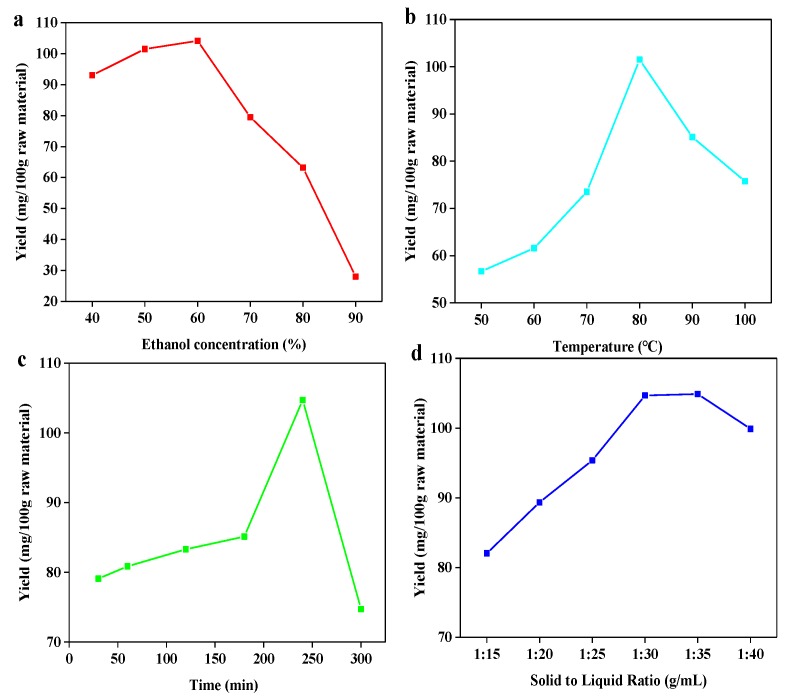
Effect of ethanol concentration (**a**); temperature (**b**); time (**c**); and solid to liquid ratio (**d**) on the extraction yield of total flavonoids.

Implementation of ultrasonic-assisted ethanol extraction was used to extract bamboo residues flavonoids (BRF). This approach was chosen given its established alignment with “green” chemistry principles due to the ethanol as the extractive solvent, as well as its ability to achieve high efficiency of flavonoid recovery. Ethanol as the extraction solution presents additional advantages due to its recyclability and generally low degree of toxicity. Extraction yield is dependent on several factors, including temperature, time, ethanol concentration and solid to liquid ratio [27]. Based on the breadth of potentially influential factors, it was important to determine the optimal conditions to design a whole extraction process for bamboo residues. To begin, a single factor designed experiment (Figure 1) followed by an orthogonal designed experiment were carried out.

As shown in Figure 1, it was found that flavonoid extraction yield increased with the rise of four parameters (temperature, time, ethanol concentration and solid to liquid ratio) before achieving the peak value and then decreased. As shown in Figure 1a, the highest extraction yield (104.167 mg/100 g bamboo residues) was obtained at ethanol concentration of 60%. The yield decreased rapidly as ethanol concentration proceeded over 60%. One possible explanation for this observation could be that increasing ethanol’s proportion in the extraction solvent progressively lowers solvent polarity, which can impede flavonoid glycoside solubility given both their high polarity and abundance in bamboo residues [28]. Regarding the temperature’s influence (Figure 1b), as temperature increased from 50 to 100 °C, the extraction yield progressively increased until reaching a maximum at 80 °C. However, further increasing the temperature resulted in lowered extraction yields. The initial improvement to yield at elevated temperatures could be explained by the combination effect of heating and ultrasonic cavitation synergistically elevating the extent of swelling of materials, which led to an improvement in extraction yield [29]. However, extraction yield decreased with temperature rising, which can be explained that the degradation of heat-sensitive flavonoids might be occured when temperatures over 80 °C [30]. With regards to extraction time (Figure 1c), a notable increase in the extraction yield was observed in the range of 30 to 240 min. However, yield began to drop in a linear fashion beyond that time point, suggesting that the decomposition of flavonoids might take place over these extended extraction periods. Finally, on the matter of solid–liquid ratio (Figure 1d), flavonoid extraction yields were observed to be the greatest at the ratio of 1:30. Further increasing solid-liquid ratio resulted in unchanged and then decreased yield, which is possible that the soluble flavonoids has been extracted thoroughly except those that are insoluble at ratio of 1:30. Based on all of the above results, it can be assessed that the optimal conditions amongst the four parameters tested are as follows: ethanol concentration of 60%, temperature of 80 °C, time of 240 min and solid to liquid ratio of 1:30, which resulted in the extraction yield of flavonoid with 104.667 mg/100 g bamboo residues. The result was similar to the work reported that the highest yield was obtained at 1:20 solid to liquid ratio using 60% methanol solution for 2 h [31].

To further optimize extraction parameters, a *L*_9_ (3^4^) orthogonal test was designed with its details presented in Table 1. Results obtained from the orthogonal test are shown in Table 2. According to the range analysis, it can be found that extraction temperature had the most significant impact on extraction yield. To verify this result, the, an analysis of variance (ANOVA) was performed to evaluate the statistical significance of process parameters. As shown in Table 3, compared to the critical *F*α value of 4.26, it can be clearly seen that temperature is the most significant. This result is consistent with the results in orthogonal experiments. In order of importance following temperature were ethanol concentration, solid to liquid ratio, and finally extraction time. The optimal extraction combination was A_1_B_1_C_2_D_3_, which differed from the results obtained by the single factor experiment. This phenomenon could be attributed to the interaction between parameters and other relevant factors unconsidered in single factor test. Under this optimal condition, the extraction yield of flavonoid can reach to 104.760 mg/100 g bamboo residues, which was similar to the result of single factor extraction experiment.

### 3.2. The Component Analysis of BRF by Liquid Chromatography-Mass Spectrometry (LC-MS)

The main component in BRF was revealed by liquid chromatography-mass spectrometry. As shown in Figure S1, the sample was separated and displayed five main peaks at 11.31, 12.64, 15.65, 24.7 and 27.71 min, respectively. Then, standards related to five peaks were ionized and results were shown in Figure S2. It can be seen that the retention times of five standards in chromatogram were in consistent with that of sample. Combined with the mass spectra data of five standards (Table S1), the peaks at 11.31, 12.64, 15.65, 24.7 and 27.71 min of BRF correspond to isoorientin, isovitexin, pinosylvin, tricin, isorhamnetin, respectively. These resulted indicated that the BRF is mainly composed of these compounds, which is in accordance to the results from bamboo leaves [32,33].

### 3.3. Optical Properties of BRF

**Figure 2:**
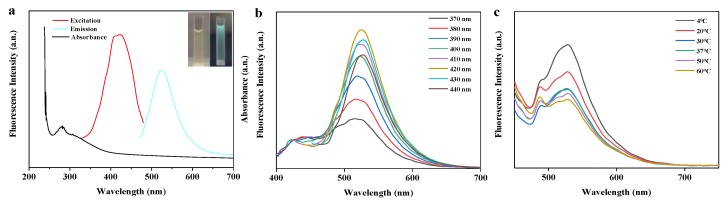
The optical properties of BRF. (**a**) Ultraviolet-visible (UV-vis) absorption, fluorescence excitation and emission spectra of BRF. Insert: images of the BRF observed under sunlight and UV light (365 nm), respectively; (**b**) fluorescence emission spectra of BRF with irradiation of various excitation wavelengths; (**c**) effect of temperature on the fluorescence intensity of BRF at the emission of 524 nm.

In general, flavonoids display characteristic light absorption in the range of 250 to 385 nm due to the absorption from the two benzene rings, namely A ring and B ring [34]. As a demonstration, ultraviolet-visible (UV-vis) absorption, fluorescence emission, excitation spectra of BRF are depicted in Figure 2a. As can be seen, the isolated flavonoids were found to have a wide UV absorption band ranging from 250 to 380 nm. The flavonoids showed a characteristic absorption peak at around 280 nm and a little shoulder at 308 nm, which can be attributed to the contribution of the π–π* transitions related to the benzoyl structure in the flavonoids [35]. In addition, a tail to the signal extended into the visible light range. In the fluorescence spectra, it can be seen that the maximum fluorescence emission peak was at 524 nm when excited at 420 nm. As seen from the inset picture, the BRF solution was visibly light yellow and transparent in daylight, while exhibiting blue-green emission irradiated by UV light of 365 nm.

Flavonoids show strong absorption peaks under UV light within wavelengths ranging from 200 to 400 nm. Thus, effect of excitation wavelength increasing from 370 to 440 nm on fluorescence emission spectra of BRF solution was investigated. As shown in Figure 2b, the emission spectra of BRF solution displayed an obvious excitation-dependent feature. When the samples were excited from 370 to 440 nm by an increment of 10 nm, the resultant fluorescence emission peak continuously red-shifted from 515 to 527 nm. The strongest emission intensity was detected at 420 nm excitation and 524 emission. Beyond this excitation wavelength the emission intensity gradually decreased. Based on identification of this maximum, all fluorescence spectra were excited at 420 nm in the following experiments.

Temperature is a crucial factor which clearly influenced both the stability of flavonoids and the accuracy of the fluorescence probe. Taking this into consideration, the effect of temperature on the fluorescence emission spectra of BRF solution at 420 nm excitation was discussed in Figure 2c. It was visualized that the fluorescence intensity continued decreasing when temperature changed from 4 °C to 30 °C, and then kept declining once temperature was over 30 °C. Interestingly, a fairly strong fluorescence intensity was observed at 37 °C. Observation of this specific fluorescence suggests that the BSF can be promising biologically when used for in vivo metal ions detection. This result is similar to a work that prepared fluorescent carbon dots, which demonstrated stability at 30 °C when temperature ranged from 3 to 60 °C [36].

### 3.4. Fluorescence Sensing of BRF Solution to Fe^3+^ Ions

#### 3.4.1. Selectivity over Various Metal Ions

**Figure 3:**
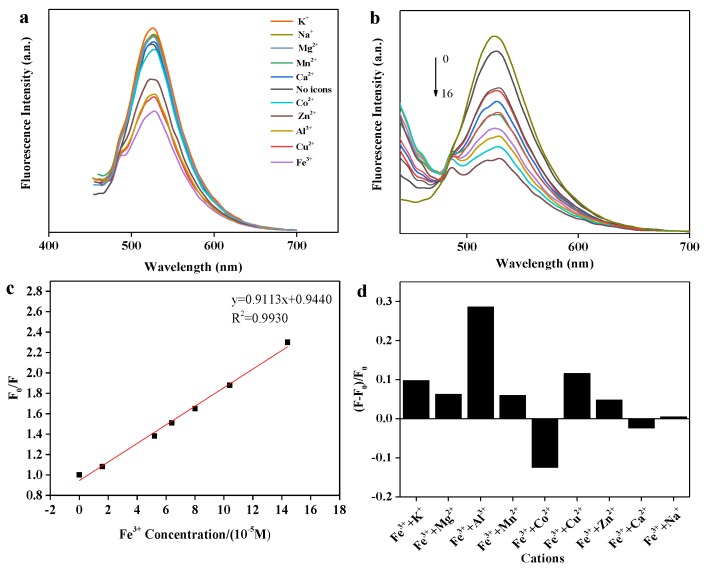
Fluorescence sensing of BRF towards Fe^3+^. (**a**) Fluorescence emission spectra of BRF with the addition of different metal ions; (**b**) fluorescence emission spectra of BRF quenched by various Fe^3+^ concentrations (0–0.16 mM); (**c**) Stern–Volmer plot for fluorescence quenching of BRF after the addition of Fe^3+^ (0–0.16 mM); (**d**) the interference of other metal ions on the fluorescence detection of BRF to Fe^3+^. F_0_ and F represent the fluorescence intensity of BRF-Fe(Ⅲ) system before and after the addition of other metal ions.

The strong binding affinity of oxygen-containing groups, such as hydroxyl and carbonyl groups on the surface of fluorescence sensor towards metal ions, which may result in remarkable changes of wavelengths and fluorescence intensity concerning emission spectra [37]. To analyze the selective fluorescence response behavior of BRF solution towards various metal ions, the emission spectra excited at 420 nm was detected, and the results are shown in Figure 3a. It can be seen that the fluorescence intensity varied with the addition of different metal ions (10 tested in total). The presence of some metal ions like Ca^2+^, Mn^2+^, Mg^2+^, Na^+^, and K^+^ contributed to the enhancement of fluorescence intensity, while other metal ions functionalized as fluorescence quencher. It has been reported that metal ions can coordinate with both hydroxyl groups and carbonyl groups on flavonoids. Resultant coordination complex then change the conjugation strength of the flavonoids, resulting in charge transfer between flavonoids and metal ions during excitation. This phenomenon is referred to as intramolecular charge transfer (ICT), which is responsible for either fluorescence quenching or enhancement [38]. Moreover, under the same conditions, a noticeable quenching behavior of Fe^3+^ to fluorescence intensity was observed compared to other metal ions, which alludes to higher thermodynamic affinity between Fe^3+^ ion and oxygen-containing groups of flavonoids [39]. This occurrence demonstrates that the BRF solution is highly selective over Fe^3+^ than other metal ions when analyzed using fluorescence methods.

#### 3.4.2. Detection and Quantification Limit of Fe^3+^ Ions

Due to the high affinity between Fe^3+^ and flavonoids, response of BRF solution towards different Fe^3+^ concentration was detected and the fluorescence spectra was shown in Figure 3b. In Figure 3b, the emission intensity diminished upon gradual addition of Fe^3+^, providing a demonstration as to the range of detection available for measuring Fe^3+^ at the tested concentrations. Furthermore, the slight red shift was observed, which can be attributed to the formation of complex between flavonoids and Fe^3+^. Flavonoids have a high super-delocalization, a complete large π conjugate system as well as a strong ligand oxygen atom and a suitable spatial configuration, which can be used as a good ligand for metal ions. Upon the addition of Fe^3+^, the interaction of Fe^3+^ with the hydroxyl group and carbonyl group of flavonoids was sufficient to the extension of conjugate π system. Then, electron delocalization of the conjugate π system increases, the transition energy decreases, causing the shift of absorption wavelength toward the long wavelength, which is so-called red shift phenomenon. In addition, a strong linear relationship (*R*^2^ = 0.99) between fluorescence intensity ratio (F_0_/F) and Fe^3+^ concentration was shown in Figure 3c. Importantly, this linear relationship is congruent with the Stern-Volmer equation:

F_0_/F = 1 + K_SV_C (3)

where K_SV_ is defined as Stern-Volmer quenching constant, C is the concentration of Fe^3+^, and both F_0_ & F are defined as fluorescence intensity of BRF solution in absence and presence of Fe^3+^, respectively. The detection limit of BRF solution was measured to be 38.0 nM, which is much lower than the maximum level (0.54 µM) of Fe^3+^ allowed in drinking water by the U.S. Environmental Protection Agency. This performance was also excellent compared to another ferrous biosensor system described in literature [40]

#### 3.4.3. Interference of Other Metal Ions on Fe^3+^ Detection

Considering the complexity of real environments which could require Fe^3+^ detection, interference by different metal ions upon the sensing system demands discussion. Changes to fluorescence intensity in a BRF-Fe(Ⅲ) system that has been spiked with different metal ions is depicted in Figure 3d. It was found that the notable interference by Al^3+^ was observed, followed by slight interference by the presence of Co^2+^, Cu^2+^ and K^+^. For other cations, however, there was a relatively slight negative impact on Fe^3+^ detection.

### 3.5. Biocompatibility Evaluation of BRF

**Figure 4:**
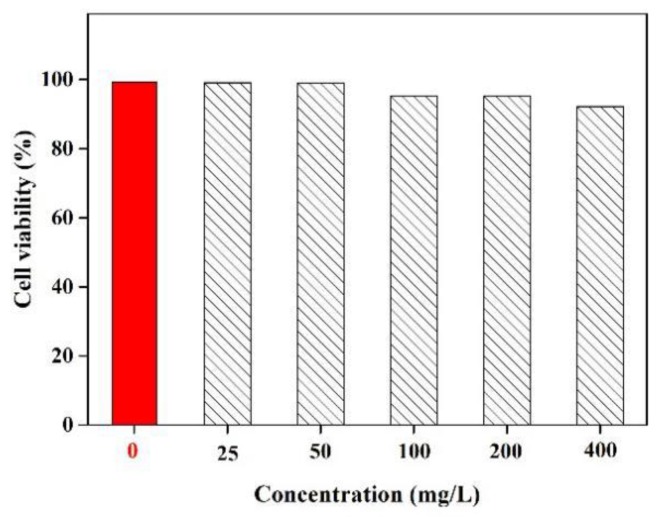
Cell viability of L929 fibroblasts after incubation with different concentrations of BRF.

During biological processes, Fe^3+^ is an important metal ion that involving oxygen uptake, oxygen metabolism and electronic transfer. The lack of Fe^3+^ can induce many serious diseases in humans, including anemia, decline of immunity and virus infection [41]. Hence, it is important to investigate the biocompatibility behavior of the prepared BRF in vitro. In this work, the in vitro cytotoxicity of BRF was estimated by using an assay involving L929 fibroblasts, with assay results shown in Figure 4.

Notably, it can be seen that cell viability remained greater than 95% when the concentrations of BRF increased from 25 to 400 mg/L. This demonstration indicates that they have excellent biocompatibility, providing further proof that they are suitable and nontoxic for detecting the Fe^3+^ when it carried out in the human body. It can be concluded that the obtained BRF with low toxicity exhibited both high sensitivity and selectivity towards metal ions and in particular, ferrous ions. Hence, the properties of biocompatibility and bioavailability of BRF allow it to be applied as a fluorescent sensor for Fe^3+^ detection in cells.

### 3.6. Intracellular Determination of Fe^3+^ by BRF

**Figure 5:**
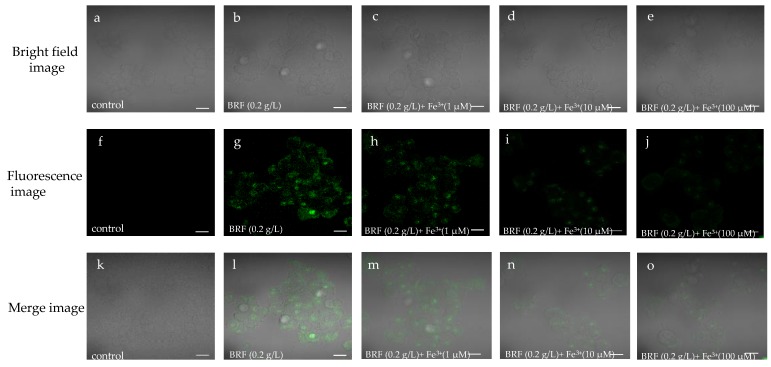
Confocal images of L02 cells. (**a**)–(**e**): bright field images; (**f**)–(**j**): fluorescence images; (**k**)–(**o**): merge images.

Considering the excellent fluorescence sensing of Fe^3+^ and biocompatibility of BRF, the bioimaging application of BRF for Fe^3+^ detection was investigated in living cells of liver cell L02 cells, which play important role in detoxification, metabolic regulation and energy storage. As shown in Figure 5, viable and intact cell morphology can be observed from the bright field and merge images, indicating the good biocompatible performance of BRF in L02 cells. Figure 5f,g showed the L02 cells did not emit fluorescence while cells with BRF incubation showed strong green fluorescence in the same condition, indicating the good cell membrane permeability property of BRF. In Figure 5h–j, it can be seen that the green fluorescence intensity of cell was decreased when it was incubated with the increased concentrations of Fe^3+^, indicating the efficient fluorescence quenching behavior of Fe^3+^ towards BRF in vitro. It can be concluded that the ICT detection system is capable of detecting Fe^3+^ in L02 cells, indicating the ability of BRF probe for Fe^3+^ detection in the biological system.

### 3.7. Antioxidant Activity of BRF

**Figure 6:**
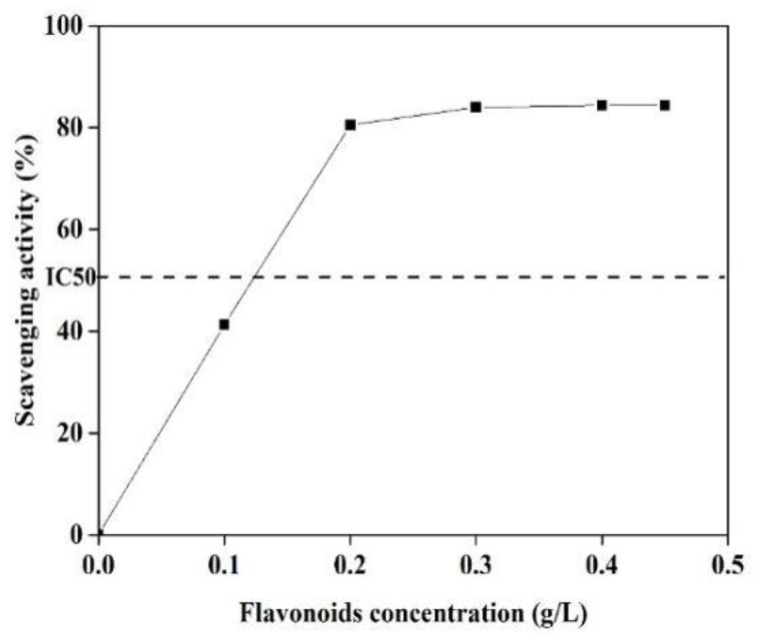
DPPH free radical scavenging ability of BRF with different concentrations of flavonoids.

It has demonstrated that C-glycoside flavonoids in bamboo extracts are mainly responsible for the biological activity, including the antioxidant activity [42]. The antioxidant potency of BRF was measured using the DPPH free-radical scavenging assay, with the results shown in Figure 6.

As it can be seen that when BRF concentration ranged from 0 to 0.30 g/L, the scavenging activity of the extracts increased from 40% to 85%. Then, the scavenging activity maintained constant when concentration was furthered increased to 0.45 g/L. The highest scavenging activity with IC50 was obtained at the concentration of 124 mg/L, which is lower than the IC50 value from 137 to 260 mg/L for the five native Brazilian bamboos [43]. This excellent antioxidant activity indicates that the obtained BRF might have additionally beneficial antioxidant capacity when it applied as the fluorescence sensing for Fe^3+^ ions.

### 3.8. The Antioxidant Effect of BRF on Hydrogen Peroxide-Induced ROS Generation in Vitro L02 Cell and in Vivo Zebrafish

**Figure 7:**
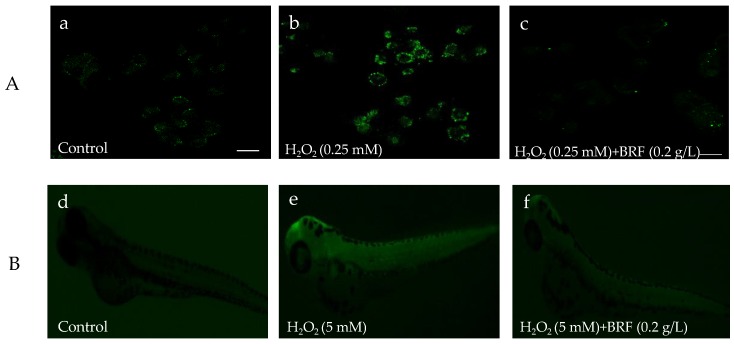
The antioxidant effect of BRF on H_2_O_2_-induced reactive oxygen species (ROS) production in zebrafish embryos. (**A**) L02 cell under fluorescence microscope; (**B**) zebrafish embryo under fluorescence microscope.

In the biological system, ROS includes H_2_O_2_, ^1^O_2_, O_2_^•−^, ClO^−^, ONOO^−^ and •OH radicals. ROS balance plays key role in protecting DNA, proteins, and lipids in the living cells. To investigate the antioxidant ability of BRF on the scavenging ROS, the level of intracellular ROS after being treated with or without BRF in zebrafish and L02 cell were detected using a chemical fluorescence method (2,7-dichlorofuorescin diacetate, DCFH-DA). As shown in Figure 7a–c, the fluorescence of L02 cell with H_2_O_2_ stimulation was notably increased compared to the normal L02 cell (control), This suggested the ROS was generated in cells by treated with H_2_O_2_. However, when these cells were incubated with BRF, the fluorescence of cell was visibly decreased. In addition, the investigation on protective effect of BRF against overproduced ROS in vivo zebrafish is shown in Figure 7d–f. Notably, the whole-body fluorescence intensity of zebrafish was significantly increased when it was treated by H_2_O_2_, which is due to the generation of ROS. It is interesting to note that the intensity fluorescence of H_2_O_2_-induced zebrafish declined, indicating the generated ROS in zebrafish was scavenged by BRF. These results suggested the excellent scavenging effect of BRF for H_2_O_2_-induced ROS generation in vivo and in vitro. It has been demonstrated that bamboo leaf flavonoids extracts could show ability to alleviate oxidative stress in HepG2 cells that were induced by oleic acid [44].

## 4. Conclusions

In this work, a sustainable flavonoid-based fluorescence sensor was successfully prepared from bamboo residues using a non-destructive extraction method. The flavonoids exhibited obvious luminescence performance with strong blue-green light at 360 nm, an expected optical property of the compounds. In addition, the flavonoids showed high sensitivity and selectivity towards Fe^3+^ detection in the range of 0 to 16 mM (*R*^2^ = 0.99) with a detection limit as low as 38 nM. The flavonoids sensor showed good bioimaging of Fe^3+^ detection in L02 cell as well as excellent compatibility and antioxidant activity to scavenge ROS produced in zebrafish and L02 cell. This robustness offers a simple and green fluorescence sensor for Fe^3+^ detection. Overall, the results show the obtained flavonoids from bamboo residues with excellent antioxidant ability can potentially be used as a metal ions sensor in the fields of bioimaging and biosensing.

## Figures and Tables

**Table 1:** Factors and levels of *L*_9_ (3^4^) orthogonal experiment.

Level	Factor			
	Solid to Liquid Ratio (A)	Ethanol Concentration (B)	Time (C)	Temperature (D)
	(g/mL)	(%)	(min)	(°C)
1	1:25	50	180	70
2	1:30	60	240	80
3	1:35	70	300	90

**Table 2:** Design and results of *L*_9_ (3^4^) orthogonal experiment.

Number	A	B	C	D	Total Flavonoids Yield mg/100g Bamboo Residues
1	1	1	1	1	84.246
2	1	2	2	2	74.504
3	1	3	3	3	96.370
4	2	1	2	3	100.942
5	2	2	3	1	68.024
6	2	3	1	2	64.221
7	3	1	3	2	76.855
8	3	2	1	3	76.016
9	3	3	2	1	74.413
k_1_	85.040	87.348	74.828	75.561	
k_2_	77.729	72.848	83.286	71.860	
k_3_	75.761	78.334	80.417	91.109	
R	9.278	14.500	8.459	19.249	

**Table 3:** Results of variance analysis.

Factor	SS ^a^	Df ^b^	MS ^c^	F ^d^	P ^e^
A	286.814	2	143.407	1.061	>0.05
B	321.580	2	321.580	2.739	>0.05
C	111.026	2	111.026	0.736	>0.05
D	625.993	2	625.993	8.150	<0.05

*F*_0.05_ (2,9) = 4.26; ^a^: sum of squares of deviation from mean; ^b^: degree of freedom; ^c^: mean square; ^d^: *F*-ratio; ^e^: percentage of contribution.

## Supplementary Materials

Supplementary materials can be accessed at: https://www.mdpi.com/2073-4360/11/9/1377/s1. Figure S1. HPLC chromatogram of the BRF detected at 280 nm; Figure S2. LCMS profiles of five standard compounds. From (a) to (e) are isoorientin, isovitexin, pinosylvin, tricin, isorhamnetin; Table S1. Main flavonoids compounds in BRF identified using LCMS profiling.
