# Supplementary material for: Isolation of the Flavonoid from Bamboo Residues and Its Application as Metal Ion Sensor in Vitro
Source: Polymers (Basel). 2019 Aug 22;11(9):1377. doi: 10.3390/polym11091377 (PMC6780781; doi:10.3390/polym11091377)
Supplement: Supplementary file 1 [file polymers-11-01377-s001.pdf]

Supplementary Materials

Figure S1. HPLC chromatogram of the BRF detected at 280 nm.

Figure S2. LCMS profiles of five standard compounds. From (a) to (e) are isoorientin, isovitexin, pinosylvin, tricin, isorhamnetin.

Table S1. Main flavonoids compounds in BRF identified using LCMS profiling.

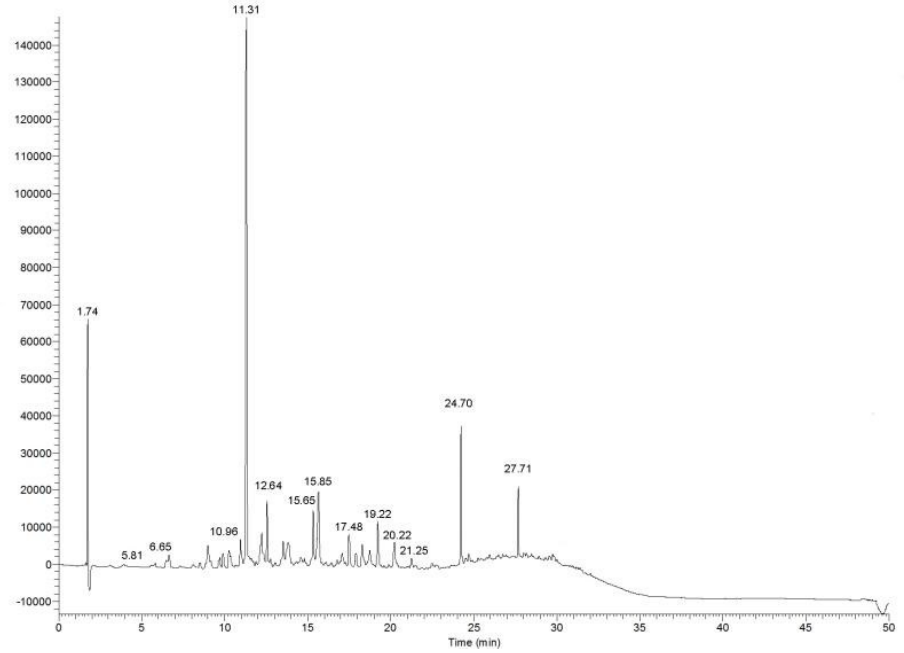
11.31
1.74
24.70
27.71
12.64
15.85
15.65
17.48
19.22
20.22
21.25
10.96
5.81
6.65
140000
130000
120000
110000
100000
90000
80000
70000
60000
50000
40000
30000
20000
10000
0
-10000
0
5
10
15
20
25
30
35
40
45
50
Time (min)

Fig. S1.

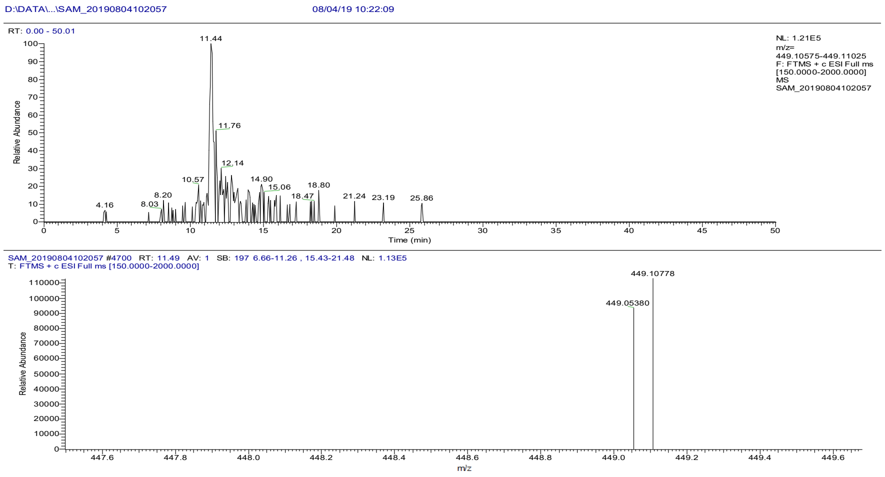
D:\DATA\...\SAM_20190804102057
08/04/19 10:22:09
RT: 0.00 - 50.01
NL: 1.21E5
m/z= 449.10575-449.11025
F: FTMS + c ESI Full ms [150.0000-2000.0000]
MS
SAM_20190804102057
11.44
11.76
12.14
10.57
14.90
15.06
18.80
8.20
18.47
21.24
23.19
25.86
8.03
4.16
Relative Abundance
Time (min)
SAM_20190804102057 #4700 RT: 11.49 AV: 1 SB: 197 6.66-11.26 , 15.43-21.48 NL: 1.13E5
T: FTMS + c ESI Full ms [150.0000-2000.0000]
449.10778
449.05380
Relative Abundance
m/z

a

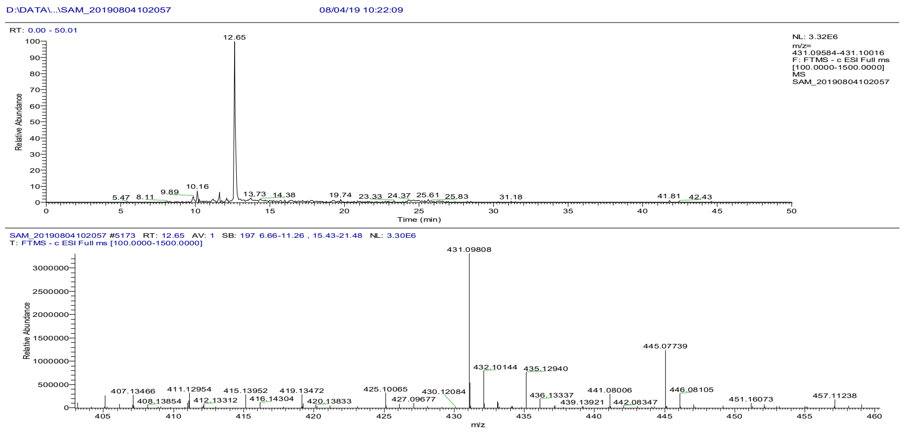
D:\DATA\...\SAM_20190804102057
08/04/19 10:22:09
RT: 0.00 - 50.01
NL: 3.32E6
m/z= 431.09584-431.10016
F: FTMS - c ESI Full ms [100.0000-1500.0000]
MS
SAM_20190804102057
12.65
10.16
9.89
5.47
8.11
13.73
14.38
19.74
23.33
24.37
25.61
25.83
31.18
41.81
42.43
Relative Abundance
Time (min)
SAM_20190804102057 #5173 RT: 12.65 AV: 1 SB: 197 6.66-11.26 , 15.43-21.48 NL: 3.30E6
T: FTMS - c ESI Full ms [100.0000-1500.0000]
431.09808
445.07739
432.10144
435.12940
407.13466
411.12954
415.13952
419.13472
425.10065
430.12084
436.13337
441.08006
446.08105
408.13854
412.13312
416.14304
420.13833
427.09677
439.13921
442.08347
451.16073
457.11238
Relative Abundance
m/z

b

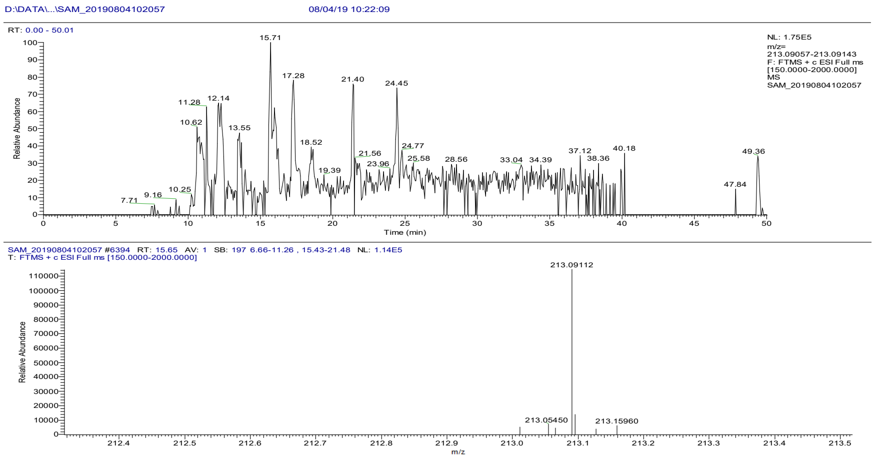
D:\DATA\...\SAM_20190804102057
08/04/19 10:22:09
RT: 0.00 - 50.01
NL: 1.75E5
m/z= 213.09057-213.09143
F: FTMS + c ESI Full ms [150.0000-2000.0000]
MS
SAM_20190804102057
Relative Abundance
Time (min)
SAM_20190804102057 #6394 RT: 15.65 AV: 1 SB: 197 6.66-11.26 , 15.43-21.48 NL: 1.14E5
T: FTMS + c ESI Full ms [150.0000-2000.0000]
213.09112
213.05450
213.15960
m/z

c

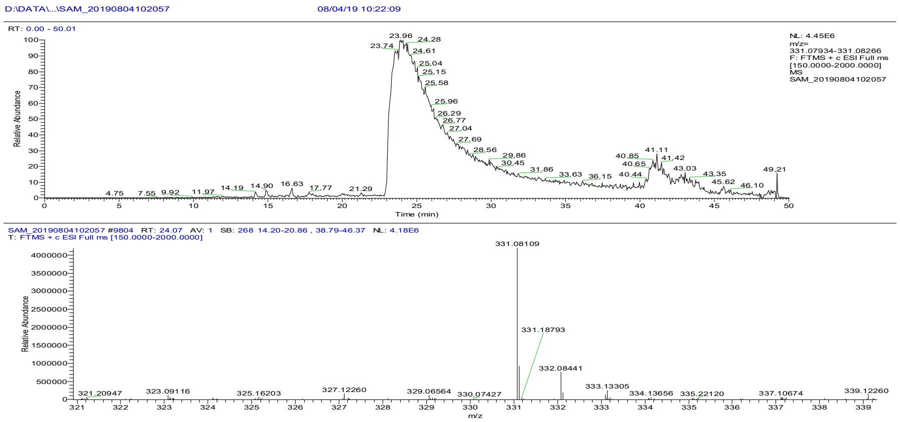
D:\DATA\...\SAM_20190804102057
08/04/19 10:22:09
RT: 0.00 - 50.01
NL: 4.45E6
m/z= 331.07934-331.08266
F: FTMS + c ESI Full ms [150.0000-2000.0000]
MS
SAM_20190804102057
Relative Abundance
Time (min)
SAM_20190804102057 #9804 RT: 24.07 AV: 1 SB: 268 14.20-20.86 , 38.79-46.37 NL: 4.18E6
T: FTMS + c ESI Full ms [150.0000-2000.0000]
331.08109
331.18793
332.08441
m/z

d

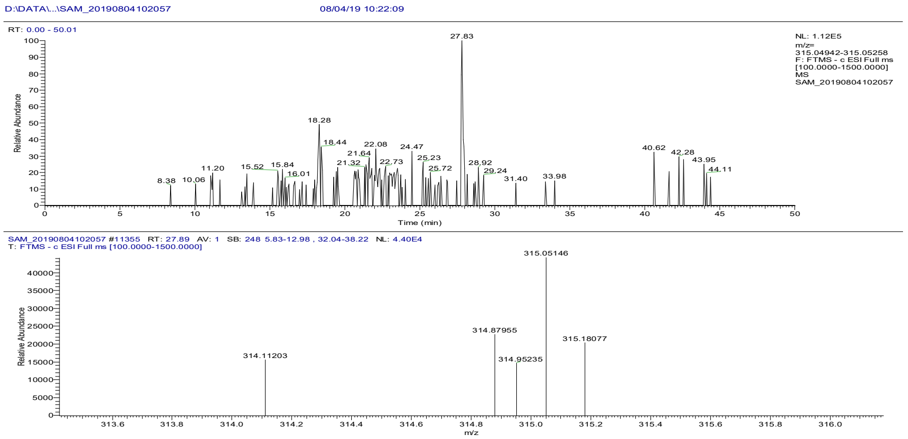
D:\DATA\...\SAM_20190804102057
08/04/19 10:22:09
RT: 0.00 - 50.01
NL: 1.12E5
m/z= 315.04942-315.05258
F: FTMS - c ESI Full ms [100.0000-1500.0000]
MS
SAM_20190804102057
Relative Abundance
Time (min)
SAM_20190804102057 #11355 RT: 27.89 AV: 1 SB: 248 5.83-12.98 , 32.04-38.22 NL: 4.40E4
T: FTMS - c ESI Full ms [100.0000-1500.0000]
315.05146
314.87955
315.18077
314.95235
314.11203
m/z

e

Fig. S2.

Table S1.

Sample	Compounds	Retention Time (RT)	Molecular weight
BRF	isoorientin	11.31	448.376
	isovitexin	12.64	432.377
	pinosylvin	15.65	212.24
	tricin	24.7	330.29
	isorhamnetin	27.71	316.27
standards	isoorientin	11.49	448.108
	isovitexin	12.65	432.098
	pinosylvin	15.71	212.091
	tricin	24.07	330.081
	isorhamnetin	27.89	316.051
